# Involvement of Dopamine D2 Receptors in Addictive-Like Behaviour for Acetaldehyde

**DOI:** 10.1371/journal.pone.0099454

**Published:** 2014-06-13

**Authors:** Anna Brancato, Fulvio Plescia, Rosa Anna Maria Marino, Giuseppe Maniaci, Michele Navarra, Carla Cannizzaro

**Affiliations:** 1 Department of Sciences for Health Promotion and Mother and Child Care “G. D'Alessandro”, University of Palermo, Palermo, Italy; 2 Department of Drug Sciences and Products for Health, University of Messina, Messina, Italy; Chiba University Center for Forensic Mental Health, Japan

## Abstract

Acetaldehyde, the first metabolite of ethanol, is active in the central nervous system, where it exerts motivational properties. Acetaldehyde is able to induce drinking behaviour in operant-conflict paradigms that resemble the core features of the addictive phenotype: drug-intake acquisition and maintenance, drug-seeking, relapse and drug use despite negative consequences. Since acetaldehyde directly stimulates dopamine neuronal firing in the mesolimbic system, the aim of this study was the investigation of dopamine D2-receptors' role in the onset of the operant drinking behaviour for acetaldehyde in different functional stages, by the administration of two different D2-receptor agonists, quinpirole and ropinirole. Our results show that acetaldehyde was able to induce and maintain a drug-taking behaviour, displaying an escalation during training, and a reinstatement behaviour after 1-week forced abstinence. Acetaldehyde operant drinking behaviour involved D2-receptor signalling: in particular, quinpirole administration at 0.03 mg/kg, induced a significant decrease in the number of lever presses both in extinction and in relapse. Ropinirole, administered at 0.03 mg/kg during extinction, did not produce any modification but, when administered during abstinence, induced a strong decrease in acetaldehyde intake in the following relapse session. Taken together, our data suggest that acetaldehyde exerts its own motivational properties, involving the dopaminergic transmission: indeed, activation of pre-synaptic D2-receptors by quinpirole, during extinction and relapse, negatively affects operant behaviour for acetaldehyde, likely decreasing acetaldehyde-induced dopamine release. The activation of post-synaptic D2-receptors by ropinirole, during abstinence, decreases the motivation to the consecutive reinstatement of acetaldehyde drinking behaviour, likely counteracting the reduction in the dopaminergic tone typical of withdrawal. These data further strengthen the evidence that acetaldehyde may play a crucial role as mediator of ethanol's central effects.

## Introduction

The neurobiological mechanism underlying the pharmacological properties of ethanol is complex and not completely elucidated [1]. In this regard, acetaldehyde (ACD), its first metabolite, has been increasingly recognized as strongly involved in various ethanol neuropharmacological, neurobiological and behavioural effects [2]–[8]. Despite its reputation as an aversive substance for long time [9], ACD possesses motivational and reinforcing properties, highlighted in rodents by different behavioural paradigms, as place conditioning [10], [11] and operant self-administration [12]–[15]; protocols which include reinstatement and conflict procedures have also been reported [16], [17].

Dopamine (DA) plays a prominent role in the different stages of the addiction cycle [18]–[20]. The dopaminergic (DAergic) transmission represents the neurobiological substrate of the acute reinforcing properties of the drugs of abuse [21] and of their enhanced incentive salience [22]. However, the function of the mesolimbic DA system is severely impaired upon cessation of the chronic exposure to several drugs of abuse, including ethanol [23]. Acute withdrawal is associated with an increase in reward thresholds in animals, a finding which mirrors the decreased activity of the mesolimbic dopamine system observed by electrophysiological recordings and *in vivo* microdialysis [24]–[27]. Furthermore a decrease in the number and function of D2 receptors, observed both in animals [28], [29] and in humans [30]–[33], is consistent with the hypodopaminergic state in ethanol withdrawal, and is functionally correlated to the enhancement in drug craving, drug intake and relapse [34]–[36].

Several reports clearly show that pharmacological properties of ACD involve the DAergic system: ACD is able to increase the neuronal firing of DA neurons in the ventral tegmental area [37], to stimulate DA release from their projections [38]–[40] and to promote, in DA terminal areas, the induction of early-gene protein expression, as c-Fos, considered as a general marker of neural activity [41]. Although this general evidence points to the involvement of mesencephalic DA neurons in ACD neuropharmacological action, the few studies exploring the neurobiological mechanisms underlying the reinforcing and addictive-like properties of oral ACD, have focused on endocannabinoid [17] and opioid [42] systems, while DA's direct contribution to ACD operant drinking behaviour still remains elusive.

We now hypothesize that ACD-induced operant behaviour can be modulated by the direct manipulation of DAergic signalling; we verify our hypothesis by investigating the role of two different D2 receptor agonists, quinpirole and ropinirole, in distinct functional phases of ACD operant-drinking behaviour. D2 receptors have both pre- and post-synaptic localization [43]–[46]: quinpirole, at low doses, has been reported to preferentially bind to D2 autoreceptors [47], [48]. Ropinirole, a post-synaptic D2 agonist, is already used to restore dopaminergic tone in Parkinson's Disease [49], [50], as well as in the normalisation of the behavioural responses to natural rewards in anhedonic states [51]. The effectiveness of these two different D2 receptors agonists in reducing drug seeking and drug taking, during extinction and relapse in ACD-induced operant behaviour, can imply important translational consequences concerning the pharmacological treatments of alcohol addiction.

## Materials and Methods

### Ethics Statement

The experimental procedures have been carried out in accordance with the Italian legislation D.L. 116/1992 and the EU Directive 2010/63/EU, dealing with research on experimental animals, and approved by the Committee on the Ethics of Animal Experiments of the University of Palermo. All efforts were made to minimize animal suffering.

### Animals

Adult male Wistar rats (Harlan, Udine, Italy) (n = 44), weighing 250–300 g, were used in this study. Rats were housed two per cage (standard rat cages, Tecniplast, Italy) with free access to food, (standard rodent diet 4RF18, Mucedola, Italy) and tap water, and kept under controlled environmental conditions (12 h light/dark cycle, temperature 22±2°C, humidity 55±10%). Animals were allowed to habituate to facilities and daily handled before initiation of the experiments, in order to reduce and minimize animal's distress. During operant conditioning experiments, they were water-restricted and allowed to drink 1 hour/day at the end of the experimental sessions. Water intake was recorded. For each experiment, animals were randomly assigned to the different experimental groups.

### Drugs

ACD 99,98% (Sigma-Aldrich SRL, Milan Italy) was stocked at −20°C and the solution was prepared with distilled water daily. ACD concentration (3.2%; 1.6 ml in 50 ml of solution) was controlled and measured with UV spectrum analysis, either at the beginning or at the end of the operant session. The procedure confirmed that ACD concentration was preserved along the 20-minute experimental procedure [16].

Quinpirole hydrochloride (Sigma-Aldrich, Milan, Italy), used in Experiment 1, was dissolved in saline solution (0.9% NaCl), and administered intraperitoneally (i.p.) (0.03 mg/kg), 30 minutes prior to start behavioural procedures.

Ropinirole (Sigma-Aldrich, Milan, Italy), used in Experiment 2, was dissolved in saline solution (0.9% NaCl), and administered daily during the 7-day deprivation 2 period (0.03 mg/kg, i.p.).

### Operant Apparatus

The experimental sessions were carried out in a custom-built operant-conditioning chamber (30×28×37 cm), placed in a dim-illuminated, ventilated, sound-attenuating cubicle. The chamber was equipped with one active lever and a cup that collected liquid from a corked reservoir, aiming at the preservation of ACD solution from evaporation, with a solenoid-actuated delivery system. It assured the delivery of 0.028 ml of solution for each lever press. Animal performance was recorded on a counter connected to the chamber. The devices were thoroughly cleaned before the introduction of each animal to ensure that the particular rat's behaviour was not affected by the detection of another rat's scent.

### Open Field Arena

Locomotor activity was measured in an Open Field with an automatic video-tracking system, Any Maze (Ugo Basile, Italy). The Open Field is a square box, 44 cm long, 44 cm wide, and 20 cm high. The software produces a quali-quantitative mapping of the motor pattern and measures Total Distance Travelled (TDT; cm) along 5 min.

### Experimental procedures

#### Timeline

The operant drinking behaviour paradigm includes consecutive experimental sessions: shaping and training (30 days); deprivation 1 (7 days); relapse 1 (5 days); extinction (1 day); deprivation 2 (7 days); relapse 2 (5 days).

#### Shaping and Training

Animals (n = 44) were shaped to lever press to obtain water on a continuous reinforcement schedule (fixed ratio 1, FR-1), until they reached the same level of performance. Afterwards training started and rats orally self-administered ACD solution in the operant chamber (FR-1) along 30 days. For each tapping, the system delivered 0.028 ml of 3.2% ACD solution. The number of lever presses was automatically recorded; ACD intake was measured by multiplying the number of lever presses by 0.028 ml, and this value corresponded to the amount of liquid missing from the reservoir at the end of each session. Animals were tested each day at the same time (9:00 to 14:00).

#### Deprivation 1

During the first 7-day deprivation period, rats were left undisturbed in their home cages and received water and food ad libitum. ACD self-administration was suspended to achieve a forced abstinence.

#### Relapse 1

After the first deprivation period, rats were exposed again to lever pressing in the operant chamber with a FR-1 response schedule, for 5 days. Responses were then recorded during the 20 min-experimental sessions. ACD solution intake was also recorded.

#### Experiment 1: administration of quinpirole during extinction and relapse 2

After the first relapse paradigm, the effect of quinpirole on ACD operant drinking behaviour was tested during extinction and relapse 2.

#### Extinction

Animals underwent an operant responding session during which reward delivery was withheld, in order to test the effect of the pharmacological manipulation on ACD-seeking behaviour. The number of lever presses throughout the 20-min session was tracked. During extinction, rats were administered with 0.03 mg/kg quinpirole (Q-group, n = 8), or vehicle (Vh-group, n = 8), 30 minutes before entering the operant chamber.

#### Deprivation 2

ACD self-administration was suspended again for 7 days to achieve a forced abstinence. Rats were left undisturbed in their home cages and received water and food *ad libitum*.

#### Relapse 2

During this phase, the two experimental groups (n = 8 each) were not treated at relapse day 1, in order to have a baseline value of drinking following abstinence. From day 2 to day 4, they were administered i.p. with 0.03 mg/kg quinpirole, or vehicle, according to their group, 30 minutes prior starting the operant session. Administrations were suspended during session 5.

The dose of Quinpirole was selected because of its capability to reduce ethanol self-administration in an analogous procedure [47]. In order to test the specificity of quinpirole effect on the operant response, a water-drinking group (n = 8) was used and subjected to the same experimental procedures than ACD-drinking rats.

#### Experiment 2: administration of ropinirole during extinction and deprivation

After the first relapse paradigm, the effect of ropinirole on ACD operant drinking behaviour was tested during the extinction and relapse 2 phases.

#### Extinction

Animals (n = 20) underwent an operant responding session during which reward delivery was suspended, in order to test the effect of the pharmacological manipulation on ACD-seeking behaviour. The number of lever presses at the end of the 20 min session was recorded. During extinction, rats were administered with 0.03 mg/kg ropinirole (R-group, n = 10) or vehicle (Vh-group, n = 10), 30 minutes before getting in the operant chamber.

#### Deprivation 2

During this session the distinct groups received ropinirole (0.03 mg/kg, i.p.), or vehicle, once daily at the same time (11:00 a.m.) along 7 days.

#### Relapse 2

During this phase animals received no administration. The number of lever presses and the amount of ACD consumed was measured after each 20-min session.

#### Open Field Test

Quinpirole and ropinirole effects on locomotor activity were also tested in the Open Field Test. Rats (n = 36) were administered with quinpirole (0.03 mg/kg, i.p.), ropinirole (0.03 mg/kg, i.p.) or vehicle, 30 minutes before entering the open field arena. Total distance travelled (TDT) at the end of the 5-minute session was measured.

### Statistical analysis

A one-way analysis of variance (ANOVA) for repeated measures was conducted in order to evaluate the effect of time on the number of lever presses during training and relapse, while a two-way ANOVA for repeated measures was employed to assess the effect of time and treatment on the number of responses emitted during relapse. Furthermore, in order to compare ACD intake during the different periods of training, and the effect of quinpirole and ropinirole treatments on ACD-seeking behaviour during extinction, and on locomotor activity, a two-tailed Student's t-test for unpaired measures was used. When necessary, simple main effects and post hoc comparisons were calculated with Bonferroni post-test (α = 0.05). Differences were considered statistically significant if p<0.05. Data are reported as mean ± SEM.

Statistical analysis was conducted by using the GraphPad Prism software, v.6.1 (GraphPad Software, San Diego, CA, USA) on data from all experimental animals used.

## Results

### Operant Self-Administration

#### Training Period

**Figure 1:**
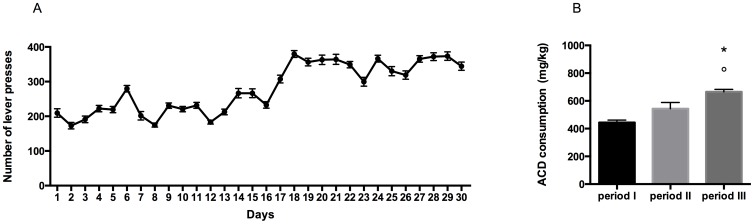
Operant drinking-behaviour pattern. Number of lever presses during the 30 days of training in the operant-drinking paradigm (A). ACD consumption (mg/kg) during subsequent periods of training in the operant-drinking paradigm. °p<0.001 Vs period I; *p<0.05 vs period II (B).

Rats readily acquired 3.2% ACD oral self-administration within the Training period, progressively increasing the number of lever presses along time. The results of a one-way ANOVA for repeated measures, showed a significant effect of time on the number of responses emitted (F_(29, 1015)_ = 68.02, p<0.0001) (Fig.1A).

In particular, during the first 10 days of exposure to ACD, rats showed a lower number of lever presses, and consequently an average intake of 444,1±124,3 mg/kg. In the second ten days of the paradigm, ACD rats' drinking behaviour increased, displaying a higher number of lever presses and greater liquid intake, and an average intake of 543,2±144,8 mg/kg. During the last 10-day period of training, rats' lever presses for ACD increased significantly with respect to the previous days, reaching a mean value of 665,3±118,8 mg/kg.

When ACD intake within the three subsequent training periods was analyzed by a two-tailed Student's t-test for paired measures, a significant difference in the amount of ACD consumed in period III with respect to period I and period II (t = 7.705, df = 9, p<0.001; t = 2.470, df = 9, p<0.0356) was observed (Fig.1B).

No differences were observed in body weight and water intake during the free-drinking hour at the end of the experimental sessions, along the 30-day training period between the two groups.

#### Relapse 1

Following the first 7 days of abstinence from ACD self-administration, rats were tested in the operant chamber to assess whether deprivation could influence their drinking behaviour. A one-way ANOVA for repeated measures showed a statistically significant effect of days on the number of responses emitted (F_(4, 140)_ = 6.188, p = 0.0001). Bonferroni post-hoc analysis evidenced a significant increase in number of lever presses in day 2 with respect to day 3 (t = 4.789, df = 140, p<0.001) and day 5 (t = 3.483, df = 140, p<0.01).

### Experiment 1: administration of quinpirole during extinction and relapse 2.

#### Extinction

**Figure 2:**
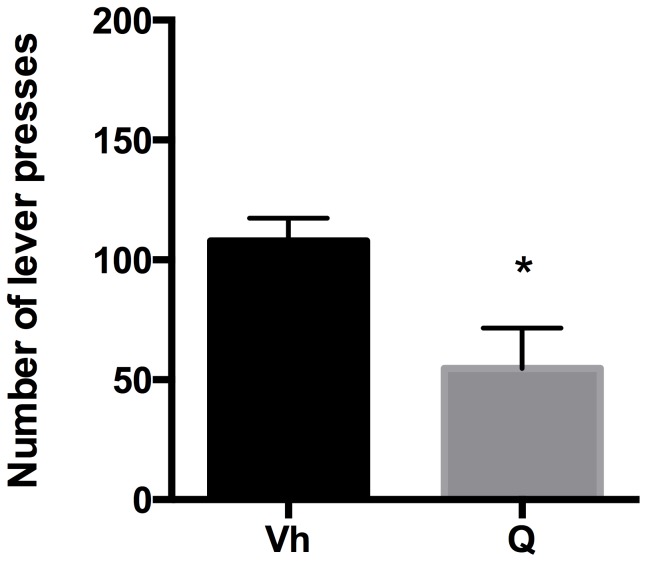
Quinpirole and Extinction. Number of lever presses during the 20-min Extinction session, in vehicle (Vh) - and quinpirole (Q) treated rats. *p<0.05 Vs Vh.

Rats were tested in the operant condition paradigm to assess quinpirole effect on drug seeking when reward delivery was suspended. The effect of quinpirole on the number of non-rewarded lever presses was analyzed by a two-tailed Student's t-test for unpaired measures. Our data indicated that quinpirole administration induced a significant reduction in the number of lever presses (t = 2.740, df = 14, p = 0.0160) with respect to vehicle along the 20 min experimental session (Fig. 2).

#### Relapse 2

**Figure 3:**
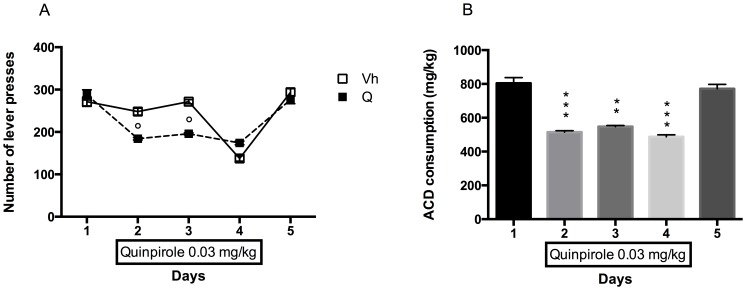
Quinpirole and Relapse. Number of lever presses in Vh and Q groups during Relapse following 1 week abstinence. °p<0.001 Vs Vh (A). ACD consumption (mg/kg) during Relapse following 1 week abstinence. **p<0.01; ***p<0.001 Vs Vh (B).

The results of a 2-way ANOVA for repeated measures including "Quinpirole Treatment" as the between-subjects factor and “Days” as within-subjects factor showed a significant effect of time, treatment, and their interaction on the number of responses emitted, F_(4, 56)_ = 88.05, p<0.0001; F_(1, 14)_ = 26.10, p = 0.0002; F_(4, 56)_ = 18.95, p<0.0001. Bonferroni post hoc analysis showed that quinpirole was able to induce a reduction in the number of lever presses with respect to vehicle in 2 out of 3 days of administration: day 2 (t = 5.883, df = 70, p<0.0001), day 3 (t = 6.991, df = 70, p<0.0001); the two ACD-drinking groups showed no statistically significant differences in days 1 and 5, when quinpirole was not administered (Fig. 3A).

Quinpirole effect was also analyzed within the treated group day by day. The results of a one-way ANOVA for repeated measure, including "quinpirole treatment" as the between columns factor, showed a statistically significant effect of the drug on the amount of ACD consumed (F_(1.490, 10.43)_ = 60.60, p<0.0001). Bonferroni post hoc analysis showed that quinpirole administration decreased the amount of ACD ingested in day 2 (t = 7.933, df = 7, p<0.001); 3 (t = 6.736, df = 7, p<0.01) and 4 (t = 9.242, df = 7, p<0.001) with respect to day 1, and also when compared to day 5, when quinpirole administration was suspended (day 2: t = 8.311, df = 7, p<0.001; day 3: t = 7.504, df = 7, p<0.01; day 4: t = 9.994, df = 7, p<0.001). No significant difference in mg/kg of ACD consumed was observed between day 5 and day 1 (Fig. 3B).

When quinpirole was administered in controls, i.e. water-drinking rats, the results of a 2-way ANOVA for repeated measures showed no significant effect on the number of lever presses emitted along the experimental days (F_(4,24)_ = 1.405, p = 0.2623).

### Experiment 2: administration of ropinirole during extinction and deprivation 2.

#### Extinction

**Figure 4:**
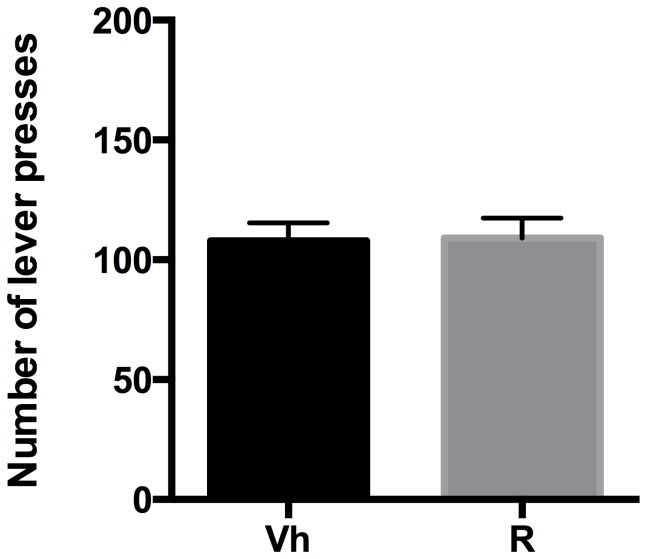
Ropinirole and Extinction. Number of lever presses during the 20-min Extinction session, in vehicle (Vh) - and ropinirole (R) treated rats.

Rats were tested on the operant condition paradigm to assess ropinirole effect on drug seeking when reward delivery was suspended. The effect of ropinirole treatment on non-rewarded lever presses was analyzed by a two-tailed Student's t-test for unpaired measures. Our data indicated that ropinirole treatment, 30 minutes before the extinction session, was ineffective and no significant difference in the number of lever presses was observed (t = 0.09720, df = 18, p = 0.9236), compared to vehicle (Fig.4).

#### Relapse 2

**Figure 5:**
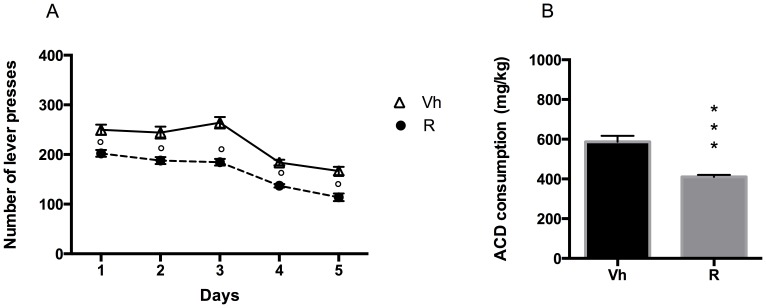
Ropinirole and Relapse. Number of lever presses in Vh and R groups during Relapse following 1 week abstinence. °p<0.001 Vs Vh (A). ACD consumption (mg/kg) during Relapse following 1 week abstinence. ***p<0.001 Vs Vh (B).

Following 7 days of abstinence from ACD self-administration in deprivation 2, rats were administered daily with 0.03 mg/kg ropinirole, and were tested again in the operant chamber to assess whether the treatment could influence their drinking behaviour in relapse 2. The results of a 2-way ANOVA for repeated measures including "Ropinirole treatment" as the between-subjects factor and “Days” as within-subjects factor, showed a significant effect of time, treatment, and their interaction on the number of responses emitted (F_(4, 72)_ = 105.2, p<0.0001; F_(1, 18)_ = 36.11, p<0.0001; F_(4, 72)_ = 2.926, p = 0.0267). Bonferroni post hoc analysis showed that ropinirole-treated group displayed a decrease in the number of lever presses in all days of relapse, on day 1, 2, 3, 4 and 5 (t = 4.047, df = 90, p = 0.0005; t = 4.800, df = 90, p<0.0001; t = 6.774, df = 90, p<0.0001; t = 3.991, df = 90, p = 0.0007; t = 4.516, df = 90, p<0.0001) when compared to vehicle (Fig. 5A).

Accordingly, a two-tailed Student's t-test for unpaired measures showed a significant reduction in the amount of ACD in ropinirole -treated group, with respect to vehicle (t = 5.617, df = 18, p<0.001) (Fig. 5B).

### Open Field Test

**Figure 6:**
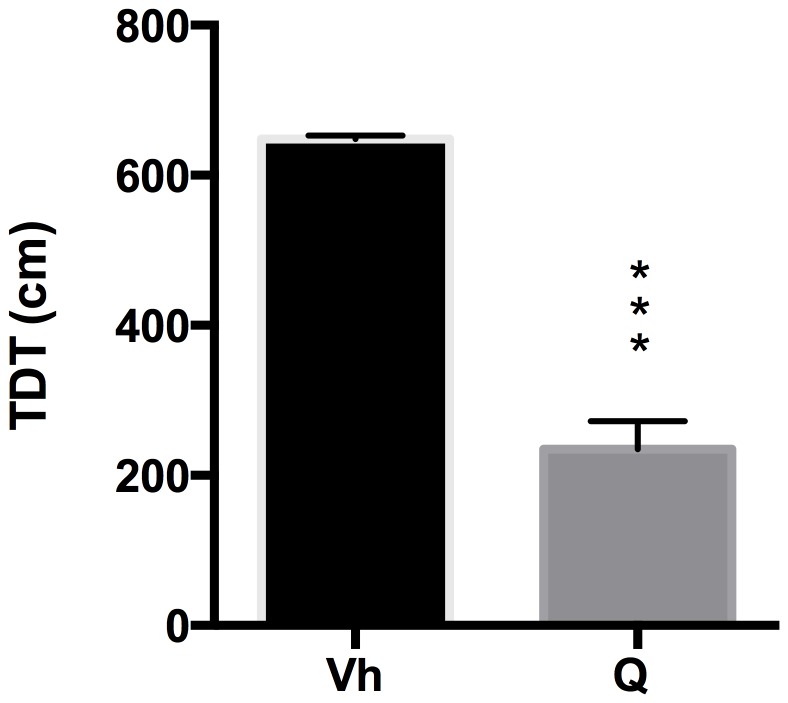
Quinpirole and OF. Effect of quinpirole on the total distance travelled (TDT) in the open field arena. ***p<0.001 Vs Vh.

**Figure 7:**
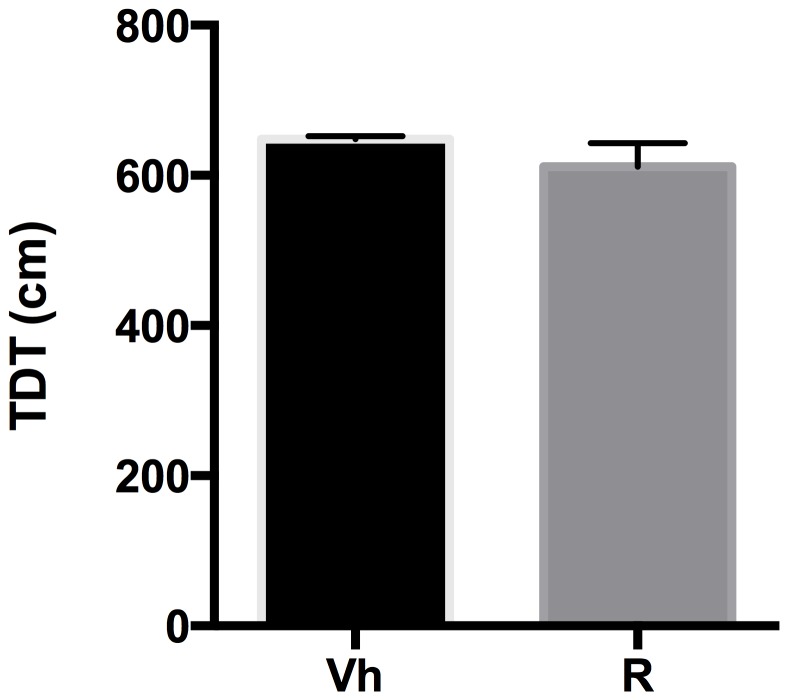
Ropinirole and OF. Effect of ropinirole on the total distance travelled (TDT) in the open field arena.

The effects of quinpirole and ropinirole treatment on locomotion in terms of TDT in the Open Field Test were analyzed by a two-tailed Student's t-test for unpaired measures. Our data indicate that quinpirole administration induced a significant reduction in TDT (t = 10.93, df = 14, p<0.0001) with respect to vehicle (Fig. 6). Ropinirole administration induced no variation in TDT (t = 1.147, df = 18, p = 0.2665) with respect to vehicle (Fig. 7).

## Discussion

The aim of the present study was to investigate the neuropharmacological basis underpinning discrete aspects of operant drinking behaviour for ACD in male rats.

Previous self-administration studies demonstrated that ACD possesses its own reinforcing and motivational properties [10], [15], [52], [53] since it is able to induce and maintain an operant behaviour in rats and promotes different drug-related behaviours, such as resilience to extinction, induction to relapse and to compulsive-like behaviour [12], [16], [17]. Recent works by Karahanian and colleagues elegantly demonstrated that ACD has a crucial role in mediating ethanol reinforcement in the VTA. Indeed, reducing ACD generation, or increasing its metabolism in the VTA, can lead to a marked reduction of ethanol intake in naive rats [5], [54], but the increase in ACD metabolism in VTA failed to affect ethanol intake in animals that consumed ethanol chronically for 2–3 months [54]. In this regard, a role for high ACD peripheral levels, able to cross the blood-brain barrier, cannot be ruled out. Since chronic ethanol exposure leads to CYP2E1 induction and decreased activity of aldehyde dehydrogenase [55]–[58], it is worth exploring the pharmacological potential properties of peripheral ACD, which may also account for its positive reinforcing effects. Remarkably, some of the behavioural features of orally self-administered ACD are sensitive to the pharmacological modulation of the cannabinoid CB1 receptor [17], as well as of the opioid neurotransmission [42]. These systems are largely involved in the induction of alcohol drinking behaviour and relapse [59], [60], [35] and can likely influence ACD drinking behaviour through the modulation of the DAergic reward pathway, thus causing DA release in the nucleus accumbens [61].

In the current experiments, the induction of ACD drinking behaviour was acquired along 30 days. Our data show that in the last period of training, rats' ACD intake was significantly higher than in the previous weeks suggesting that the incentive motivation for the substance had also been increasing along time [62]. Operant conditioning is a behavioural paradigm specifically tailored to reflect the measure of the reinforcing properties of self-administered drugs [63]–[68]. Chronic exposure to relative high ACD concentrations, as those used in the present study (3.2%), aims at creating conditions for ACD central accumulation, since this small molecule can be found in the central nervous system when administered i.p. at doses of 100 mg/kg [69], [70]. According to our data, ACD intake reached mean values of 665,3 mg/kg, within the 20 min-operant session, and it is conceivable that such chronic and increasing intake of ACD can overwhelm the metabolic barrier constituted by epithelial aldehyde dehydrogenase (ALDH1), a low Km ACD-oxidizing enzyme expressed in gastrointestinal tract [71]. Systemic absorption after ACD oral ingestion has been already demonstrated by previous studies on ACD self-administration by Peana and colleagues [12]. Therefore, increasing blood ACD concentrations could saturate the moderate aldehyde dehydrogenase activity of BBB capillaries [72]–[74], [8], enter the brain, and exert central activity.

Our results are in agreement with previous data from this laboratory [16], and can be ascribed to the rewarding and motivational properties of ACD. Animals easily self-administer ACD [13]–[16], likely as a consequence of its reinforcing properties, reported to be 1000-fold stronger than ethanol's [15], [53]. Although apparently in opposition with reports on primarily ACD aversive effects following acute peripheral administration [75], our results must be interpreted in the context of the particular experimental protocol. ACD operant-drinking behaviour is induced and maintained along a relatively long period, which is likely required for exerting pharmacologically significant central effects. On the other hand, our findings are in line with early studies reporting positive euphoric effects following moderate consumption of ethanol in subjects treated with aldehyde dehydrogenase inhibitors, such as disulfiram [76].

DA involvement in the operant behaviour for ACD was assessed by using quinpirole and ropinirole, DA D2 receptor-selective ligands with a different pharmacodynamic profile, whose administration aimed at selectively modulate the DAergic synapse in different functional states. Indeed, the rationale of the experiment was that quinpirole, administered during extinction and relapse, could phasically inhibit DAergic signalling, as a consequence of its activity as agonist at D2 receptors in the presynaptic terminal, and reduce thus drug-seeking behaviour; ropinirole, on the other hand, as a D2-D3 receptor agonist, was administered daily during the deprivation period in order to stimulate the DAergic post-synaptic terminal, thus reducing the craving for the substance during reinstatement.

Our data clearly show that, when tested during extinction, quinpirole acute administration was able to decrease the number of lever presses, when compared with vehicle. Lever pressing is thought to reflect learned processes related to motivation to seek the substance [77] and it is to be considered as a measure of appetitive “ACD seeking-like” behaviour, which is in turn related to the induction of mesolimbic DA release [61].

Our evidence shows that quinpirole, at the doses used in this study, is responsible for the decrease in the number of lever presses through the reduction in DA neural firing, obtained by the activation of D2 pre-synaptic receptors [45], [46], indicating that ACD-seeking behaviour directly depends on DA neurotransmission.

In order to assess the role of dopamine neurotransmission on the reinstatement of ACD consumption following a period of forced abstinence, quinpirole was administered during relapse. In particular, along the relapse experiment, animals were administered with quinpirole during day 2, 3 and 4, in order to consider day 1 as a reference of the baseline drinking behaviour, and to evaluate drinking behaviour restoration, once the administration ceased at day 5.

Quinpirole was able to decrease the number of responses emitted and, induced a significant reduction in ACD intake, within the quinpirole treated group, when compared with the first and the last relapse days. Accordingly, the dose of quinpirole used in these experiments has previously been shown to decrease ethanol-reinforced responding [47], likely by disrupting dopamine transmission, *via* pre-synaptic receptors stimulation [48], [78], [79].

It has long been known that activation of D2-like receptors hyperpolarizes DA neurons and inhibits their firing activity [80]–[82]. Consistent with these data, anatomical studies revealed that D2-like receptors are predominantly expressed on the dendrites of DA neurons, where their inhibitory activity plays a predominant role [83]. Quinpirole, activating D2 pre-synapting autoreceptors, appears to dissociate the process of primary reinforcement from processes regulating instrumental response initiation, maintenance, and selection [84], hence leading to a decrease in ACD-attributed salience, and consequently in the motivation to work for drug self-administration.

In agreement with previous studies [85]–[87] and consistently with the pre-synaptic action exerted, quinpirole reduced locomotor activity in terms of total distance travelled, when tested in the Open Field. Nevertheless, no differences in rats' behavioural reactivity in the operant chamber were observed during the operant self-administration sessions, ruling out a non-specific effect of the drug in the reduction of the operant behaviour. Indeed, when tested on the operant-drinking behaviour for water, quinpirole did not exert any effect, with respect to vehicle, highlighting a specific activity on motivation for ACD.

A different activity on the DAergic synapse, i.e. a post-synaptic receptor modulation, was achieved by ropinirole administration. Ropinirole is a well-tolerated, selective D2-D3 agonist used in improving the motor symptoms of Parkinson's Disease, hence able to increase DA neurotransmission [49], [86], [88]. Due to its post-junctional activity, ropinirole was administered during extinction in order to verify whether it was able to affect drug-seeking behaviour. Our results show that ropinirole failed to induce any modification in lever pressing. This result is consistent with ropinirole activity in promoting dopamine signalling, but since ropinirole is devoid of abuse liability, it was not able, in our experimental conditions, to modify motivation when lever pressing was not associated to reinforcement.

The acute administration of low dose-ropinirole did not induce significant variations in total distance travelled in the Open Field, when compared to vehicle, confirming a mild D2-D3 receptor stimulation. On the contrary when high dose-dopamine agonists are employed, long-lasting alterations in DAergic functions appear. These are associated with increased locomotor activity and enhanced DA overflow in brain limbic areas, defined as behavioural sensitization [89], [90].

Abrupt withdrawal from alcohol and other substances of abuse is associated with a decrease in DAergic neuronal activity [24]–[27], [91], [92]. This effect has been indicated as contributing to the dysphoric state of abstinence [36], and to the enhancement in drug intake during relapse [23], [34]. Moreover, decreased levels of D2-D3 receptors in the striatum can be visualized by brain imaging in abstinent alcoholics: this feature has become a common marker of addiction in human patients [30], [32], [93].

On the other hand, manipulation of the D2 receptors seems effective in reducing drug-intake and relapse: when rodents receive viral-mediated gene transfer of D2 receptors to the nucleus accumbens, they display attenuated ethanol consumption [94].

The extent to which a mild D2-D3 stimulation, during withdrawal, can influence the reinstatement behaviour for ACD, was thus investigated.

Once acquired a long habit to ACD self-administration, and in order to be tested for reinstatement, rats underwent forced abstinence, during which they were daily administered with a low dose of ropinirole. The subsequent testing for relapse shows that ropinirole was able to significantly decrease the number of lever presses for ACD, with respect to vehicle, along the whole relapse phase. Ropinirole administration during abstinence could provide a mild stimulation of post-synaptic D2-D3 receptors, overcoming the dysfunctional reduction in DA neurotransmission associated with craving and vulnerability to relapse. Our data are in line with the hypothesis that long lasting ACD self-administration, and related behaviours, can induce a hypo-DAergic tone during withdrawal, as already demonstrated for alcohol and other addictive substances [23], likely induced by a dysfunctional inhibition of DA VTA neurons [95].

Recent reports demonstrate that ropinirole is able to reduce the subjective reinforcing effect of cocaine in humans [96], [97]; moreover Hoefer et al. [51] showed that ropinirole was able to counteract the reward deficit in methamphetamine withdrawal in rats, supporting the hypothesis that activating, rather than blocking, the DAergic system could help attenuating drug seeking behaviour [98]. Additional studies need to be undertaken to evaluate ropinirole efficacy in alcohol relapse in order to suggest its administration as a pharmacological aid in controlling the chronic relapsing nature of alcohol abuse.

## Conclusions

Taken together, these findings show that the pharmacological modulation of DA synapse has a direct impact on ACD-related behaviour. Animals do self-administer ACD, and its oral consumption, or the associated conditioning context and cues, exerts a DA-releasing effect, as suggested by the different sensitivity to distinctly acting D2 agonists. Manipulation of the D2 signalling represents a promising strategy to finely tune DA control over alcohol and ACD actions in the brain. Post-synaptic D2 receptors are involved in the modulation of ACD-induced phenotypes, since their mild and subchronic activation during withdrawal is able to affect the subsequent drug seeking and intake during relapse, Moreover, ropinirole treatment during abstinence has face validity within the clinical setting, and this enhances the translational relevance of preclinical animal testing for medications in treating substance use disorders.
